# Reversible inactivation of the transcriptional function of P53 protein by farnesylation

**DOI:** 10.1186/1472-6750-6-26

**Published:** 2006-05-29

**Authors:** Bettina Couderc, Marie Penary, Mustapha Tohfe, Anne Pradines, Antoine Casteignau, Danièle Berg, Gilles Favre

**Affiliations:** 1 INSERM U563, Department Innovations thérapeutiques et Oncologie moléculaire, Institut Claudius Regaud and Faculté des Sciences Pharmaceutiques, Toulouse, France

## Abstract

**Background:**

The use of integrating viral vectors in Gene therapy clinical trials has pointed out the problem of the deleterous effect of the integration of the ectopic gene to the cellular genome and the safety of this strategy. We proposed here a way to induce the death of gene modified cells upon request by acting on a pro-apoptotic protein cellular localization and on the activation of its apoptotic function.

**Results:**

We constructed an adenoviral vector coding a chimeric p53 protein by fusing p53 sequence with the 21 COOH term amino acids sequence of H-Ras. Indeed, the translation products of Ras genes are cytosolic proteins that become secondarily associated with membranes through a series of post-translational modifications initiated by a CAAX motif present at the C terminus of Ras proteins. The chimeric p53HRCaax protein was farnesylated efficiently in transduced human osteosarcoma p53-/- cell line. The farnesylated form of p53 resided mainly in the cytosol, where it is non-functional. Farnesyl transferase inhibitors (FTIs) specifically inhibited farnesyl isoprenoid lipid modification of proteins. Following treatment of the cells with an FTI, p53HRCaax underwent translocation into the nucleus where it retained transcription factor activity. Shifting p53 into the nucleus resulted in the induction of p21^waf1/CIP1 ^and Bax transcription, cell growth arrest, caspase activation and apoptosis.

**Conclusion:**

Artificial protein farnesylation impaired the transcriptional activity of p53. This could be prevented by Farnesyl transferase inhibition. These data highlight the fact that the artificial prenylation of proteins provides a novel system for controlling the function of a transactivating factor.

## Background

One of the common obstacles encountered in gene therapy trials is the potential deleterious effect of the integration of the ectopic gene to the cellular genome. As an example a serious adverse event after successful gene therapy for X-linked severe combined immunodeficiency has been described with a LMO2-associated clonal T cell proliferation in two patients [1,2]. A way to eradicate this negative effect is to induce the death of the modified cells upon request including a suicide gene in the gene transfer vector. Previous approaches used gancyclovir-induced cell death post transduction with a viral vector containing a Herplex Simplex Virus-Thymidine kinase expression cassette [3]. However the effectiveness of this strategy may be blunted because of their more limited effect on quiescent or slowly dividing cells that require prolonged expression of the therapeutic gene and long term administration of the prodrugs. Another way to induce the death of gene modified cells is to promote expression of a pro-apoptotic protein, a cytotoxic protein or a drug sensitive inducer protein such as CD20 as suggested recently [4] via a pharmacological control of the transgene transcription [5,6]. Transcription regulation is usually achieve by means of cell-permeant-inducing agents such as tetracycline, macrolides, oestrogen, progesterone, isopropyl-b-D-thiogalactoside and ectysone [7,8]. Here we proposed a post translational control of a protein. We studied a way to pharmacologically induce protein function upon request by reversible sub-cellular localization of the protein.

Protein prenylation is required for the biological functions of several proteins by permitting association with the cell membranes and encouraging protein-protein interactions with other regulatory molecules. Protein isoprenylation is a post translational isoprenoid lipid modification of substrate proteins by isoprenic lipids [9]. 0.5 to 1 % of cellular proteins are isoprenylated (for review[10]), including members of the RasGTPase superfamily, several protein kinases and phosphatases, and a variety of proteins involved in nuclear integrity and centromere function [10-12]. Two type of enzymes catalyse protein isoprenylation, the CAAX prenyl transferase, farnesyl transferase (FTase) and Geranylgeranyl transferase I (GGTaseI) that recognize CAAX (A is aliphatic and X is any amino acid) C terminus peptide motif and rabGGTase or GGTaseII that recognizes CCX or CXC C terminus motifs. FTase or GGTaseI catalyse the covalent attachment of the 15 carbon farnesyl or the 20 carbon geranylgeranyl respectively to the cysteine of the CAAX motif. The terminal X residue of the CAAX motif determines whether farnesylation or geranylgeranylation occurs: FTase prefers X to be methionine, serine, alanine or glutamine, as for Ras proteins [13] while GGTaseI prefers leucine or isoleucine. There are exceptions to this general rule since RhoB can be farnesylated or geranylgeranylated *in vivo *by FTase and GGTaseI respectively [14] and since N-Ras or K-Ras but not H-Ras can be geranylgeranylated by GGTaseI when the FTase is inhibited. Protein prenylation is the first step of a complex protein processing including proteolytic cleavage of the AAX peptide, carboxymethylation of the prenylated cysteine residue, and lastly for some proteins, attachment of a palmitate residue near the prenylated cysteine. The development of FTase inhibitors (FTIs) has raised the possibility of specifically inhibiting the function of proteins involved in oncogenesis such as Ras oncoproteins. By preventing Ras farnesylation, FTIs severely impair Ras functions because of the inability of the non farnesylated protein to anchor to the membranes [15]. FTIs are currently under evaluation in phase II/III clinical trials for the treatment of cancer [16] and show relatively low-toxic effects [9].

We recently showed that a artificial three-component chimaera consisting of the ribosome recruitment core of the eIF4G1 eukaryotic translation factor, the RNA-binding domain of the R17 bacteriophage coat protein and the plasma membrane localization CAAX motif of farnesylated H-Ras can have its translational activity inhibited through protein farnesylation that could be restored by FTI treatment. This result put on light the possibility of a pharmacological switche that control gene expression at the translational level [17]. Here, we hypothezize that this concept could be applied to control the proteins that exert their functions in specific cellular compartment such as nuclear transcriptional factor as p53.

The gene encoding p53 mediates a major tumor suppression pathway that is frequently altered in human cancers [18]. P53 is inhibited during normal cell growth by MDM2 a proto-oncogene through either ubiquitin-dependent p53 degradation in the cytoplasm or repression of the transcriptional activity of p53 in the nucleus [19]. P53 is activated following cellular stresses leading to its phosphorylation and translocation to the nucleus. Nuclear activated p53 binds to specific DNA sequences and triggers the transcription of target genes thus functionning, at least in part, as a transcriptional regulator. P53 contributes to tumor suppression through at least two mechanisms, arrest of cell proliferation [20] and induction of cell death through apoptosis [21].

The aim of this study was to find a way to induce the death (here by apoptosis) of targeted cells upon request. The way that has been chosen is to control the function of a protein (here a transcriptional factor, p53) by acting on its localization. To inactivate p53 function we targeted it to the cell membranes by post translational modifications. We have generated a chimeric p53 protein with the 21 COOH term amino acids of H-Ras (HRCaax) fused to the COOH term of p53. We have demonstrated that an inactive chimeric p53HRCaax gains its cellular functions in SaOs-2 cells (activation of p21^waf1/CIP1 ^and Bax transcription, growth arrest and apoptosis) under FTI treatment. These data highlight the fact that the artificial prenylation of proteins provide a novel system for controlling the function of a transactivating proteins.

## Results

The SaOs-2 human osteosarcoma is a poorly differentiated, growth factor-insensitive cell line. This p53-/- cell line was selected because an ectopic expression of p53 induces their apoptosis [22].

Several Ad vectors encoding different mutated forms of the pro-apoptotic gene p53 were constructed to transduce these SaOs-2 cells. In this study, our aim was to compare the efficiency of mutated form of p53 versus wtp53 in inducing cellular responses, such as apoptosis only in the presence of FTI.

Adenoviral vectors have been constructed to efficiently transduce SaOs-2 cells. The proapoptotic genes were under the transcriptional control of the strong CMV promoter. We generated Adp53wt, Adp53HRCaax in which the TGA codon of the p53 coding gene has been replaced by the sequence encoding 21 COOH term amino acids of H-Ras which correspond to the membrane binding domain of H-Ras allowing the whole post translational processing of the protein, and Adp53HRSaax as a control. In this control vector, the TGA codon of the p53 coding gene has been replaced by the sequence encoding 21 COOH term amino acids of H-Ras with a single base mutation allowing the translation of a serine instead of a cysteine in the CAAX motif of the chimeric protein. This p53HRasSAAX is unable to be processed. Brand et al [23] showed that *in vitro *ΔE1 Ad vector could lead to growth retardation, prolongation of the G2/M phase and induction of apoptosis if applied at a high MOI. To distinguish between the cytotoxicity of the vectors and the effect of the transgene we compared the vectors mentioned above with either AdeGFP (enhanced Green Fluorescent Protein) or AdLuc (Luciferase). Using AdeGFP, we confirmed that more than 95% of the cells were GFP positive when using 100 physical particles/cell and no significant cytotoxicity was seen 7-days post transduction (data not shown). These data demonstrated, under our conditions, that cell responses observed bellow were due to transgene expression and that modest doses of vectors led to efficient transduction.

### Ad transduced SaOs-2 cells could express the farnesylated form of p53

**Figure 1:**
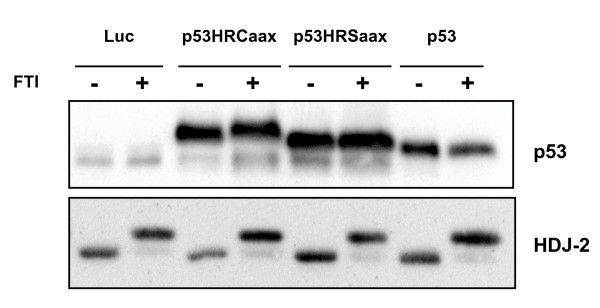
**Expression of ectopic p53 and endogenous HDJ-2 in human SaOs-2 cells**. Cells were transduced with adenoviral vectors expressing the indicated p53 proteins or luciferase as a control. Total protein extracts (80 μg) of SaOs-2 cultures transduced with different Ad-vectors and untreated (-) or treated (+) with 15 μM FTI were prepared 48 hr post transduction and analysed by immunoblotting employing the anti-p53 (1:500) and anti HDJ-2 (1:1000) antibodies.

Forty eight hr post transduction of SaOs-2 cells by the different Ad vectors, cells extracts were analyzed by Western blot (Fig. 1). As expected, AdLuc transduced cells did not express p53 protein. Following transduction with Adp53wt, Adp53HRCaax or Adp53HRSaax, p53 protein expression was observed. These results are consistent with expression of the ectopic p53 gene. All p53 mutants were expressed roughtly at the same level. Moreover, treatment of the cells with FTI did not induce any variation of the p53 expression irrespective of the form tested. Several proteins, aside from the Ras family are known to be farnesylated. These farnesylated proteins may be useful surrogate markers for determining whether a specific FTI is actually inhibiting farnesylation, by providing an indirect measure of FTase inhibition. Example of these proteins include HDJ-2, a chaperone protein. The inhibition of farnesylation of HDJ-2 can be monitored by immunoblotting, since its unfarnesylated form displays a reduced mobility in SDS-PAGE relative to its farnesylated versions [24]. When SaOs-2 transduced cells were exposed to 15 μM FTI-277, a mobility shift on SDS-PAGE was observed in HDJ-2, reflecting inhibition of FTase. Moreover, a slight mobility shift in the P53HRCaax band was noted, suggesting that FTI-277 prevents the protein farnesylation of this mutant (Fig. 1).

### Hras membrane binding domain induced p53 membranes localization

**Figure 2:**
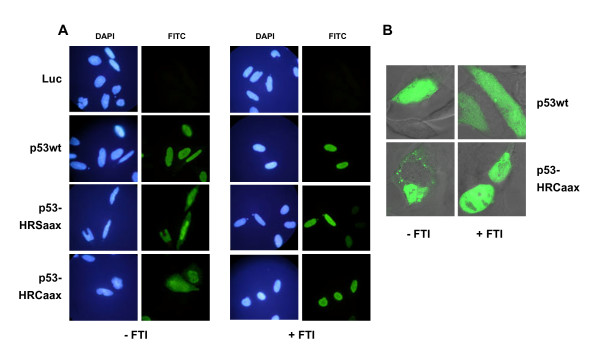
**Subcellular localization of p53 in the different transduced and FTI-treated SaOs-2 cells**. A: AdLuc, Adp53wt, Adp53HRSaax, or Adp53HRCaax transduced SaOs-2 cells were untreated or treated with 15 μM FTI for 24 hr. They were then stained with the FITC-conjugated anti human p53 antibody (1:100) and DAPI = 4',6'-diamino-2-phenylindole. The subcellular localization of p53 was examined microscopically. B: Confocal microscopy of transduced Adp53wt or Adp53HRCaax cells treated with FTI for 24 hr.

The sub-cellular localization of the different P53 mutants was analyzed 48 hr post transduction of SaOs-2 cells by the different Ad vectors (Figure 2A).

Transducing the cells with Adp53HRCaax resulted in a distinctive, punctuate staining pattern in the cytosol with a perinuclear concentration (Adp53HRCaax) excluding the nucleus. Confocal imaging confirmed that p53HRCaax was perinuclear and cytoplasmic (Figure 2B).

In the presence of 15 μM FTI, Adp53HRCaax transduced SaOs-2 cells expressed p53 exclusively in the nucleus suggesting that inhibition of farnesylation of p53 abolished its membranes sequestration.

Mihara et al [25] recently reported that a fraction of stess-induced wild-type p53 translocates to mitochondria during p53 dependent apoptosis after DNA damage and under hypoxia. We have shown here that the farnesylated form of p53 did not localize preferentially to the cell plasma membrane as expected. Therefore we checked by confocal microscopy whether p53HRCaax could localize to the mitochondria in the absence of FTI by colocalization with mitochondrial markers (mitotraker red). Just as wtp53, p53HRCaax did not translocate to the mitochondria in the transduced SaOs-2 cells either 24 or 48 hr post transduction (data not shown).

As a control, when Adp53wt or Adp53HRSaax transduced cells were stained, a nuclear localization of p53 was observed irrespective of whether the cells are untreated or treated or not with FTI (Adp53wt, Adp53HRSaax). Culture with FTI had no effect on p53 localization for these p53 forms.

### The farnesylated form of p53 (p53HRCaax) had reduced transactivation activity

**Figure 3:**
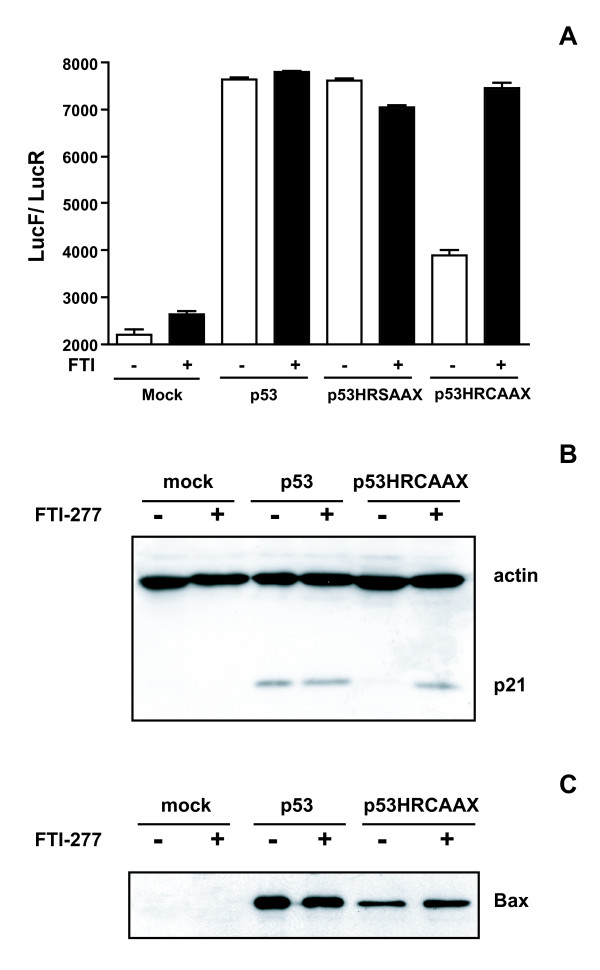
**p53-mediated transcriptional transactivation**. A : p53-mediated transcriptional transactivation as determined by luciferase activity in SaOs-2 cells co-transfected with a p53-responsive promoter luciferase plasmid and a renilia luciferase expression plasmid for transfection efficiency. Cells were then transduced with the indicated p53 adenoviral vectors. Cell extracts from each triplicate were assayed for both firefly and *Renilla *luciferases. Results are expressed as the ratio of activity of the firefly to the *Renilla *luciferase. The average mean +/- SD values and the induction factor relative to the transfection-containing no viral vector from two sets of experiments with triplicate transfections are shown. B: Relative levels of p21^waf1/CIP1 ^protein were measured by immunoblot assay using lysates (80 μg) prepared from SaOs-2 expressing luciferase (mock), p53wt, or p53HRCaax cultured for 48 hr in the presence (+) or absence (-) of the FTI (15 μM). C : Relative levels of Bax protein were measured by immunoblot assay using lysates (80 μg) prepared from SaOs-2 expressing luciferase (mock), p53wt, or p53HRCaax and cultured for 48 hrs in the presence (+) or absence (-) of the FTI (15 μM). One representative of three different experiments is shown.

The tumor suppressor activity of p53 is related to its ability to interact with both DNA and proteins. As a result of complex patterns of both DNA-protein and protein-protein interactions, expression of the down-stream genes that participate in the control of DNA replication, repair, cell cycle and apoptosis can be either elevated or decreased by p53. We have used a reporter gene assay to analyse the transcriptional activity of the different p53 mutants using the reporter vector pGL3-in which the luc reporter gene is driven by a promoter that is sensitive to induction by p53. It is anticipated that the expression of the luciferase gene will be enhanced in the presence of a functional p53. Using the polyethylenimine (PEI) method, we transiently transfected the p53-/- cells SaOs-2 with this Luc gene as reporter, plus a renilia luciferase expression plasmid to control transfection efficiency. Then, these cells were transduced with the different Ad vectors in the presence or absence of 15 μM FTI. Both LucF and LucR activities were measured. As shown in Figure 3A, p53-mediated transcriptional transactivation was effective in cells transduced with either Adp53wt or Adp53HRSaax in the presence or absence of FTI. In contrast, p53-mediated transcriptional transactivation was effectively lowered in p53HRCaax transduced cells, although FTI-277 treatment restored the transactivation efficiency of the p53HRCaax mutant.

We then analysed the induction of the p53 endogenous target genes, p21^waf1/CIP1 ^and Bax. Western blotting was performed for p21^waf1/CIP1 ^and Bax to detect functional p53 activity in cells with and without exposure to the different vectors in the presence or absence of the FTI. As expected p53wt, but not p53HRCaax transduced cells expressed p21^waf1/CIP1 ^within 48 hr of transduction (Fig. 3B). Both cells expressed Bax at different levels (Fig. 3C). FTI treatment induced p21^waf1/CIP1 ^expression or a higher level of Bax expression in p53HRCaax cells, while no modification of protein levels was observed in Adp53wt transduced cells. (Figs. 3B and 3C).

Overall these data demonstrated that the HRCaax domain fused with the p53 protein impaired the transcriptional transactivation function of the protein, although this could be reinduced by farnesyl transferase inhibition.

### FTI triggered cell growth inhibition and apoptosis of the p53HRCaax mutant

**Figure 4:**
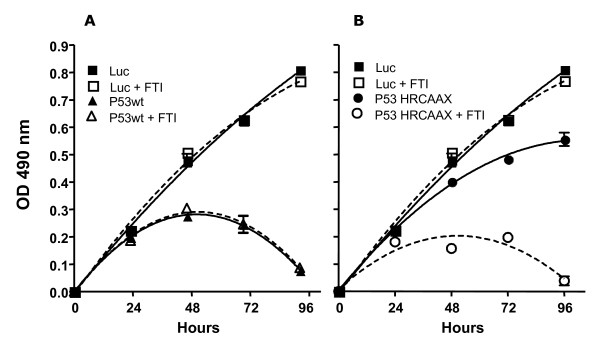
**In vitro assessment of the antiproliferative effect of the chimeric p53 genes**. Cell viability determined by colorimetric assay with MTT after transduction.

The p53 protein is a potent inhibitor of cell growth, arresting the cell cycle at several points and, under certain circumstances, activating the apoptotic machinery leading to cell death [26]. To compare the antiproliferative activity of wtp53, p53HRCaax and p53HRSaax, cell viability was determined using an MTT assay at 24, 48, 72 and 96 hr post-transduction by the different Ad vectors. SaOs-2 cells transduced with Adp53wt, Adp53HRSaax in the presence or absence of FTI or those transduced with Adp53HRCaax plus the FTI all showed a similar level of cell death. In contrast SaOs-2 cells transduced with Adp53HRCaax in the absence of the FTI were more resistant to cell death (Fig. 4).

**Figure 5:**
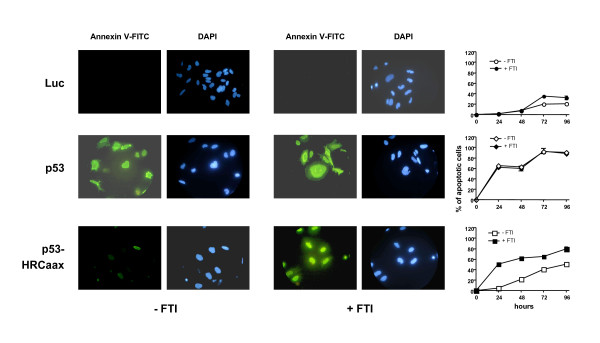
**Proportion of cells expressing markers of apoptosis 48 hr post transduction with the adenoviral vectors**. Samples of cells were stained with annexin V-FITC 48 hr post transduction to detect externalized phosphatidylserine. Apoptosis in expressing cells was quantitated by FITC staining, counting 20 random microscopic fields. One representative experiment from a total of three is shown. Percentage of apoptotic cells was determined microscopically. Values are the means +/- SD of three different experiments.

To quantify the apoptosic effects, Annexin V-positive populations were estimated by fluorescence microscopy at various times after transduction (Fig. 5). Transdution of cells with Adp53HRCaax in the absence of FTI resulted in only background levels of apoptotic cells, similar to those of mock tranduction. In contrast, Adp53HRCaax in the presence of FTI induced the apoptosis of more than 80 % of SaOs-2 cells, as seen with Adp53wt. Transduction of cells with AdLuc resulted in only a a very few apoptotic cells, such as in the non transduced cells.

Therefore, we have shown that the farnesylated form of p53 elicits no apoptosis of SaOs-2 in the absence of the FTI. Yet the ability to elicit apoptosis could be restored in the presence of the FTI.

## Discussion

We have shown that the artificial prenylation of proteins could provide a novel system for controlling the function of a protein such as here a transactivating factor. The farnesylation of a protein changed its cellular localization therefore its function was inhibited. This post translational modification could be prevented by inhibition of farnesyl transferase leading to protein activation. These new properties of an ectopic protein could be used to develop a 'gain of function' approach designed to allow the death by apoptosis of targeted gene modified cells upon request. To prove the concept we have chosen to use p53 as it is a tumor suppressor gene involved in cell cycle arrest and programmed cell death (apoptosis). The properties of p53 are mainly linked to its ability to induce the transcription of genes, such as the cyclin-dependent kinase inhibitor p21^waf1/CIP1 ^or Bax [27,28]. We constructed a p53 chimeric protein which is a substrate for farnesyl transferase by fusing the membrane binding domain of H-Ras that includes the 21 COOH term amino acids of the protein to the COOH end of p53. We used this domain because, in contrast to N-Ras and K-Ras, H-Ras membrane binding domain is not a substrate of GGTaseI when FTase is inhibited. This eliminates the possibility of protein geranylgeranylation under FTI treatment. We have demonstrated that it is the C terminus membrane binding domain of H-Ras, but not that of the CAAX box permitting only farnesylation which avoids nuclear localization of the p53 transcriptional factor (data not shown). This highlight the fact that only farnesylation is not sufficient to induce membrane attachment of a chimeric protein and that complete post translational processing is necessary for this purpose.

The post translational modification of the chimeric p53 protein is illustrated by a slight shift of the farnesylated form of the p53 protein, as observed in the Figure 1. We have verified that the non processed form of the H-Ras binding domain p53 chimeric protein does not impair its transcriptional activity. Indeed, p53HRSaax, like p53HRCaax in the presence of the FTI, was able to induce transcription of responder genes as efficiently *in vitro *as *in vivo *and was also able to induce apoptosis of the SaOs-2 cells.

P53 shuttles within the cell between the nucleus and the cytoplasm, possessing nuclear localization signals (NLS) and a nuclear export signal (NES) [29]. It was a challenge to impede the nuclear translocation of a protein containing an NLS domain and this was achieved by lipid modification which avoids nuclear localization of the p53 transcriptional factor. This highlights the possibility of developing this strategy so as to control the protein function of other nuclear proteins. We have shown here that the lipid processed form of p53 whilst located in part in the plasma membrane, was found preferentially in the cytoplasm. This observation is not surprising since Chiu et al [15] have shown that farnesylation of the CAAX motif could also target Ras proteins to the endoplasmic reticulum and Golgi membranes. In these locations they encounter Rce protease and prenylcysteine-directed carboxymethyltransferase. Thus nascent Ras proteins are present, at least transiently, on the endoplasmic reticulum and Golgi. The fact that farnesylation could control cellular localization of different chimeric protein (farnesylated form of eGFP) has also been described previously [30,31].

We have used farnesylation of p53 to control its localization and therefore it's function. We have shown here that bypassing the nucleus by targeting p53 to other cell compartments is sufficient to significantly reduce the marked apoptosis of p53-deficient tumor cells in the absence of an FTI. This therefore establishes the proof of principle that a chimeric p53 can initiate apoptosis only upon treatment of cells with the FTI,.

FTIs have previously demonstrated low toxicity in normal cells. Several FTIs are currently being evaluated in phase II and phase III clinical trials [9,32]. FTIs can exert dramatic effects on cancer cells, including morphological changes, inhibition of anchorage-independent growth and alteration of cell cycle progression. Although FTIs clearly inhibit Ras farnesylation it is unclear whether their antiproliferative effects result exclusively from their inhibition of Ras functioning [16]. FTIs have effects on several other prenylated proteins involved in crucial cellular signal transduction pathways, such as the centromere binding protein (CENP)-E and CENP-F, peroxysomal membrane and nuclear membrane (lamin A and B) associated proteins, or members of the Rho proteins family. FTIs affect the PI3-K/Akt cell survival pathway. They also inhibit soft agar growth of several breast cancer cells lines independent of their Ras mutant status, probably through an alternative target such as the protein RhoB which regulates receptor trafficking and cell adhesion/motility. In total more than 100 polypeptides possess a CAAX sequence that potentially can be farnesylated and such FTIs may have multiple targets that may be inhibited to produce a net antiproliferative effect or apoptosis [32]. As expected in the transformed SaOs-2 cellular model used in these data, few apoptotic cells have been noted following FTI treatment. However, a high percentage of apoptosis has been noted in our experiments only if FTI treated cells were previously transduced by p53HRCaax. Our results confirmed the data reported by Nielsen et al. that combination therapy with a replication-deficient recombinant adenovirus, which expresses the human p53, and an FTI have synergistic or additive antiproliferative effects on a panel of tumor cells in vitro [33].

## Conclusion

This work has been performed to find application in gene therapy protocols including, amongst others, i) the potential for inducing the activation so that the function of an ectopic protein at a defined moment and ii) the safety of therapeutic gene expression for the cells being treated. For this second purpose, integrating vectors could be constructed as a polycistronic vector. It would contain the gene of interest and the gene encoding a chimeric pro-apoptotic protein such as Bax, TRAIL or p53 such as what is currently done with the HSV-tk strategy. Upon request, if deleterious effect appears on gene modified cells such as transformation of the gene modified cells, we could apply FTI and so induce the death of the modified cells. The combination of FTI which have antitumoral effect plus the apoptosis of the newly transformed cells could allow the destruction of the unwanted transformed cells. The synergestic effect of the FTI and p53 induced apoptosis could be of greater interest than the HSV-tk strategy. Undoubtedly, the challenge now is to tease apart the intricate circuitry that controls the cellular location of pro-apoptotic-proteins.

## Methods

### Reagents

Mouse monoclonal antibody p53 (Bp53-12) was from Santa Cruz Biotechnology (TEBU, Le Perray en Yvelines, F). FITC-conjugated mouse anti-human p53, mouse anti human Bax, or p21^waf1/CIP1 ^were purchased from BD Biosciences/pharmingen (F). Rabbit anti human HDJ2 was from Lab Vision Inc, (Fremont, CA). FITC-Peroxidase-conjugated anti-rabbit and anti-mouse secondary antibodies, the β-actin antobodies and a liquid alkaline phosphatase detection kit were from Sigma (F). Mitotraker Red was obtained from Molecular Probes (Eugene, OR).

### Cell culture

The 293 (a transformed human embryonic kidney cell line) and SaOs-2 (human osteosarcoma p53-/-) cell lines were obtained from the American Type Culture Collection (Rockville, MD) and were maintained as monolayers at 37°C in a humidified 5%CO2 atmosphere in DMEM medium supplemented with 10 % FCS.

### Construction and preparation of adenoviral vectors

The different ADNc which had to be cloned into the adenoviral plasmid (pShuttle) have been obtained by PCR amplification. The forward primer used for all the PCR amplification was : 5' ATAAGAATGCGGCCGCCATGGAGGAGCCGCAGTCAG. We generated PCR fragments corresponding respectively to wt p53, P53HRCaax and P53HRSaax by using as reverse primer : 5'CCCAAGCTTTCAGTCTGAGTCAGGCCCTTCTGTCTTGAACATGAG, 5' CCCAAGCTTTCA**GGAGAGCACACACTTGCAGCTCATGCAGCCGGGGCCACTCTCATCAGGAGGGTTCAGCTTCCGCAGCTTGTG**GTCTGAGTCAGGCCCTTCTGTCTTGAACATGAG and 5' CCCAAGCTTTCA**GGAGAGCACGCTCTTGCAGCTCATGCAGCCGGGGCCACTCTCATCAGGAGGGTTCAGCTTCCGCAGCTTGTG**GTCTGAGTCAGGCCCTTCTGTCTTGAACATGAG. Replication-deficient (ΔE1, E3) adenoviral vectors expressing the different chimeric p53 or control (Adp53wt, Adp53HRCaax, Adp53HRSaax, AdLuc) under the transcriptional control of the CMV promoter were constructed with the AdEasy System (Qbiogen, F) according to the manufacturer's protocol. Initially, a PCR amplified *No*t1-*Hin*dIII fragment containing the different form of p53 was subcloned into the *No*t1-*Hin*dIII-digested pAd shuttle CMV to get shuttle vectors pAdp53wt, pAdp53HRCaax and pAp53HRSaax. Replication-defective adenoviruses (Adp53wt, Adp53HRCaax and Adp53HRSaax) plus adenoviral vectors control AdLuc (encoding luciferase) or AdeGFP (encoding enhanced Green Fluorescence Protein) were produced by transfection of 293 cells with a single isolate of each recombinant adenoviral vector, expanded, and purified as described [34]. Viral titers were initially determined by optical absorbance at 260 nm. All the viral DNA have been isolated from the viral particles, amplified by PCR and sequenced.

### In vitro adenoviral transduction

At day 0, 10^5 ^cells were seeded in a six-well plate. At day 1, one of the 6 wells was trypsinated and the cells were counted. This information was used to infect each cell line at the desired multiplicity of infection (MOI). Transductions were in 5% CO_2 _incubators at 37°C for 1 hr using 500 μl infection medium (the same medium used for each cell line + 2 % FBS) with agitation. Pilot experiments with AdeGFP determined the optimal MOI for the SaOs-2 cells. In these experiments (data not shown) all cells became GFP positive, without manifesting toxicity, for up to 7 days after infection at a MOI of 100:1.

### Immunoblotting

Cells were seeded in 35 mm dishes and grown to approx. 50 % confluence before transduction. Total protein extracts were prepared 48 hrs post transduction, by lysing the cultures in 150 μl lysis buffer containing 1% NP40, 0,1% SDS, 5 mM DTT and 1 mM PMSF for one hr on ice. Lysates were centrifuged at 13000 rpm for 5 min to remove nuclei and precipitates. Samples containing 80 μg total cellular protein were subjected to 12,5 % SDS-PAGE and transferred to a nitrocellulose membrane (Amersham Biotech, F). Membranes were blocked for 1 hr at room temperature in TBST-0.1% Tween 20/5% non-fat milk then incubated overnight with antibodies directed against β-actin (1:10,000), Bax (1:500), p53 (1:500), p21^waf1/CIP1 ^(1: 500), or HDJ2 (1:1000). For signal detection the secondary mouse or rabbit antibody was used at a dilution of 1:10,000. Finally the blots were washed and developed using enhanced chemiluminescence (Amersham Biotech, F) according to the manufacturer's protocol and exposed to radiographic films (Eastman Kodak).

### Immunofluorescence and microscopy

For fluorescence analyses cells were washed twice with PBS and fixed for 5 min at room temperature in PBS containing 3,7% formaldehyde. The cells were then permeabilized with 0,2% Triton X-100 in PBS for 5 min at room temperature. After repeated washes with PBS, cells were incubated with FITC-conjugated mouse anti-human p53 antibody or appropriate controls for 1 hr, washed twice with PBS and finally examined with a Leitz fluorescence microscope. To stain the mitochondria (MT), cells were incubated with 250 nM Mitotracker Red in pre-warmed full medium for 30 min at 37°C and then washed twice with full medium.

### Transcriptional activation

SaOs-2 cells were plated at 10^5 ^cells/35 mm tissue culture dish. 24 hr later cells that had reached 60–80% confluence were co-transfected with 1 μg p53SREluc and 3 μg preniliaLuc (rluc) reporter plasmids (PGL2 Promega). P53SRELuc plasmid contains two copies of the p53 binding site (GC3p53 element) upstream of the HSV-tk promoter cloned upstream of the DNA encoding the firefly luciferase (Luc) and a poly(A) addition signal. For all transfections plasmid DNA were mixed with JetPEI as described by the manufacturer. Six hr later the medium was replaced with fresh medium and cells were transduced by the different adenoviral vectors at a MOI of 100. All experiments were performed in triplicate. After 48 hr culture, cells were lysed in 100 μl reporter lysis buffer (Promega). Ten μl aliquots of each cell lysate were added to 90 μl luciferase assay substrate solution and reporter activity was measured using a single photo channel in a packard scintillation counter (Meriden, Conn). Each set of experiments was repeated. The relative reporter gene expression was calculated as a ratio of that with the adLuc control, arbitrarily taken as 1. The mean and SD were calculated from the two sets of experiments.

To demonstrate transcriptional activation by p53 proteins *ex vivo*, Western analysis for p21^waf1/CIP1 ^and Bax proteins was performed using monoclonal antobodies. The procedure used was as described above for p53 detection.

### Growth curves

For vector sensitivity experiments cells were trypsinized and seeded into 96 plates (10^4 ^cells per dish) and then allowed to adhere for 24 hr. Cells were then incubated with vectors for 1 hr; whilst untreated cells received an identical manipulation without vector and mock infected cells received an identical manipulation with the AdLuc vector. Cells were washed once with PBS and then fresh medium was added. Cell numbers were enumerated daily using 3-(4,5-dimethythiazl-2-yl)-5-(3-carboxymethoxyphenyl)-2-(4-sulfophenyl)2H-tetrazolium inner salt (MTT).

### Apoptosis assay

Apoptosis was evaluated quantitatively by measuring phosphatidylserine externalisation, an early apoptotic alteration. This was performed by fluorescence staining using the annexin V-FLUO staining kit (Roche, F) according to the manufacturer's recommendations. The proportion of annexin V stained cells was determined with FITC-positive cells scored using a fluorescent microscope (Olympus IX70). Images were recorded with a digital SPOT camera.

## Authors' contributions

BC, MP, MT and AC carried out the cellular studies, BC, MP and DB the synthesis of the adenoviral vectors (molecular biology and virology). BC and AP are responsible for conceiving this work. BC participated in the design, coordination and drafting of this manuscript. GF is the director of the laboratory. All authors read and approved the final manuscript.
